# Corneal and tear film immunology in anterior uveitis: protocol for a systematic review and meta-analysis

**DOI:** 10.1136/bmjopen-2026-121276

**Published:** 2026-08-12

**Authors:** Jessie L Ye, Janelle Tong, Ching Yi Wu, Alexander S Fraser, Myra B McGuinness, Lyndell L Lim, Laura E Downie

**Affiliations:** 1 Department of Optometry and Vision Sciences, The University of Melbourne, Parkville, Victoria, Australia; 2 Ophthalmology Department, Southend University Hospital, Westcliff-on-Sea, Essex, UK; 3 Centre for Eye Research Australia Ltd, Melbourne, Victoria, Australia; 4 Department of Surgery (Ophthalmology), Melbourne Medical School, The University of Melbourne, Parkville, Victoria, Australia

**Keywords:** OPHTHALMOLOGY, IMMUNOLOGY, Corneal and external diseases, Diagnostic Imaging, Inflammation

## Abstract

**Abstract:**

**Introduction:**

Anterior uveitis is an intraocular inflammatory condition that can lead to vision impairment without appropriate and timely treatment. Determining its aetiology can be difficult due to its heterogeneous clinical presentations, which challenges effective treatment. The primary aim of this systematic review is to comprehensively identify and synthesise evidence on the immunological features of anterior uveitis in the human cornea and tear film, as a foundation for considering the utility of such features as biomarkers for disease classification, prognosis and monitoring treatment responsiveness.

**Methods and analysis:**

Comprehensive electronic database searches will be performed in Ovid MEDLINE, Ovid Embase and the Cochrane Central Register of Controlled Trials (CENTRAL) without date restrictions. Two independent review authors will screen retrieved titles and abstracts in Covidence relative to predefined study eligibility criteria. Studies judged eligible or potentially eligible for inclusion will undergo full-text review using a similar method. Disagreements will be resolved by consensus. For each included study, risk of bias will be assessed using validated tools appropriate to the study design. Where sufficient and appropriate data are available, meta-analyses will be performed. For outcomes where a meta-analysis is not possible, a narrative synthesis will be provided. This review will be reported in accordance with the Preferred Reporting Items for Systematic reviews and Meta-Analyses (PRISMA) 2020 statement.

**Ethics and dissemination:**

Ethics approval is not applicable for this review as no original data will be collected. The results of this review are expected to be disseminated through a peer-reviewed publication and/or scientific conference presentations.

**PROSPERO registration number:**

CRD420261339682.

**STRENGTHS AND LIMITATIONS OF THIS STUDY**

- A broad search strategy ensures a comprehensive capture of the literature relating to human corneal and tear film immunological features in anterior uveitis.
- Risk of bias will be assessed for each included study to ensure that conclusions are appropriately framed according to the quality of the available evidence.
- The completed systematic review will be reported in accordance with the Preferred Reporting Items for Systematic reviews and Meta-Analyses (PRISMA) 2020 statement, and a meta-analysis will be performed if more than one study reports quantitative data for the same outcome.
- The absence of a universally accepted diagnostic approach for anterior uveitis means that variability in diagnostic methods and disease definitions across studies may affect the consistency of results.

## Introduction

 Uveitis encompasses a group of intraocular inflammatory disorders with a global prevalence estimated at more than 2 million cases in 2021.^1^ If inadequately managed, it may result in long-term vision impairment, due to associated ocular complications of the disease and/or treatment. Approximately 20% of uveitis cases result in legal blindness,^2–4^ and while the condition may occur at any age, it most commonly affects individuals of working age.^3 5^ Uveitis can be classified based on the anatomical location of the inflammation, with anterior uveitis being the most prevalent form.^6^ In anterior uveitis, the anterior chamber is the primary site of inflammation, which includes the iris and the pars plicata of the ciliary body. However, uveitis at a given anatomic location encompasses a broad spectrum of disease entities. Therefore, anatomical classification alone is insufficient, and the disease is often further categorised by aetiology, with terminology varying depending on the clinical or research criteria used.^7^ Accurate and timely identification of the uveitis aetiology is essential for initiating appropriate treatment. For example, a missed diagnosis of infectious uveitis (e.g., due to syphilis) could lead to the use of corticosteroids unopposed by targeted antimicrobial therapy, with the potential for poor clinical outcomes.

Anterior uveitis is clinically diagnosed based on the detection of anterior chamber inflammation during a comprehensive ophthalmic examination, after excluding intermediate or posterior segment involvement and other potential differential diagnoses (e.g., intraocular injury). Disease activity and clinical response are typically quantified using the Standardisation of Uveitis Nomenclature (SUN) Working Group^8^ grading schemes for anterior chamber cells and flare. This requires a clinician to visually estimate the degree of cell (inflammatory infiltrate) and flare (protein) load in the anterior chamber, on an ordinal scale ranging from 0 (floor) to 4+ (ceiling). Despite its widespread use and well-defined classification criteria, the SUN assessment of intraocular inflammation severity is necessarily dependent on subjective observer estimates.^9^ Measurement error tends to be greater at the extremes of the SUN scale.^10 11^ Consequently, particularly in clinical research, this has driven an exploration of more objective and automated measures of intraocular inflammation severity, including imaging modalities such as anterior segment optical coherence tomography and laser flare photometry.^12 13^ Although the SUN grading schema provides a standardised framework for defining the extent of intraocular inflammation, there are currently no clinical metrics to define a patient’s prognosis in anterior uveitis.^11 14 15^ Taken together, these limitations in current clinical assessment highlight the potential benefit of complementary, objective markers that reflect underlying ocular immune activity as a means to improve disease characterisation, monitoring and prognostication in anterior uveitis.

In the context of immune cells and the cornea, keratic precipitates (KPs) are frequently used by clinicians as diagnostic clues in anterior uveitis.^16 17^ Keratic precipitates are deposits that form on the corneal endothelium and consist of immune cell aggregates derived from the uveal vasculature that accumulate in the aqueous humour during intraocular inflammation. Visualisation of KP by researchers using high-resolution in vivo confocal microscopy (IVCM) has demonstrated utility in linking specific KP features to uveitis aetiology. Wertheim *et al* were the first to demonstrate this relationship, by correlating KP morphological characteristics with uveitis subtypes.^17^ These observations were further classified by Lim *et al*, who identified eight morphological subtypes of KP and reported that the ‘cluster’ and ‘nodular’ forms were associated with infectious uveitis.^18^ However, KP typically exhibit a variable distribution across the corneal endothelium, which can make their precise categorisation, from a two-dimensional IVCM image, challenging. Furthermore, KP morphology on both slit lamp biomicroscopy and IVCM can change from one subtype to another over the course of the disease, and with treatment.^17 19 20^ For example, granulomatous uveitis (e.g., Vogt-Koyanagi-Harada uveitis) may initially appear with fine KP that only become granulomatous when the disease turns chronic.^21^ Therefore, assessment of KP morphology using IVCM alone may not fully capture the dynamic cellular and molecular immune processes underlying uveitis, which may be necessary to achieve a reliable aetiological classification.

Recognising these limitations, Postole *et al* used IVCM to categorise immune cells in the corneal epithelium of individuals with anterior uveitis.^22^ In this study, the authors classified corneal immune cells based on their morphology, with type one as ‘dendritic-like cells’, and type two as ‘cell bodies lacking dendrites’. They reported that herpetic anterior uveitis showed a distinct pattern defined by a higher dendritic-like cell density in the central cornea; this was in agreement with the earlier findings of Knoll *et al.*^23^ Within the human cornea, the predominant immune cell populations are now recognised to include macrophages, dendritic cells and T cells.^24^ While it was originally presumed that the healthy corneal epithelium only contained dendritic cells, based on studies of mice housed under pathogen-free conditions, recent work has revealed the presence of intraepithelial lymphocytes (T cells) in the healthy human cornea.^24^ These findings were partly enabled by a novel imaging technique, called functional IVCM (Fun-IVCM), which allows for the dynamic behaviours of individual immune cells to be longitudinally evaluated through the tissue depth.^24^ The features of presumed dendritic cells in the human cornea, which likely also encompass the recently defined T cell population, have been studied under both conditions of homeostasis and in inflammatory states, using static IVCM techniques.^25–27^ Higher numbers of immune cells have been described in the human corneal epithelium in conditions characterised by inflammation.^28 29^ In addition to cell density, the morphological characteristics of corneal immune cells have been quantified.^22^ However, as traditional IVCM involves evaluating cell features using a single (static) image at a single time point, it has limited ability to reliably delineate immune cell subsets in vivo. This is where newer imaging techniques (e.g., Fun-IVCM) that enable morphological and dynamic (morphodynamic) cell analyses may yield additional insight(s).^30^ These findings highlight the need for further research and the potential benefit of adopting dynamic in vivo imaging techniques, to better understand corneal inflammatory responses. It is thus timely to undertake the present review, involving the collation and synthesis of evidence relating to how corneal immunology might be altered in anterior uveitis, and in response to treatment, as a foundation for considering how these features could serve as meaningful indicators of ocular inflammation and/or disease quiescence.

Overlying the cornea, the tear film is critical for supporting corneal function. Tear fluid has a complex composition of thousands of proteins,^31^ including immunomodulatory mediators. Tears have a mediator profile similar to the aqueous humour, making them a useful medium for biomarker studies.^32^ As intraocular fluid extraction is invasive and not feasible routinely, research has focused on leveraging tear fluid for the assessment of pro-inflammatory mediators. In uveitis, elevated tear mediators have been consistently reported, including increased levels of interleukin (IL)-8, interferon gamma-induced protein 10 (IP-10), vascular endothelial growth factor (VEGF) and transforming growth factor-beta 2 (TGF-β2),^33^ as well as IL-6 and IL-10,^15^ compared with controls. Other studies suggest potential uveitis subtype-specific tear mediator profiles, such as elevated IL-6 in Human Leukocyte Antigen B27 (HLA-B27)-associated uveitis,^34^ and higher levels of IL-8, S100 calcium-binding protein A12 (S100A12) and soluble intercellular adhesion molecule-1 (sICAM-1) in paediatric juvenile idiopathic arthritis-associated uveitis.^32^ However, a lack of large-scale studies, differences in protein analysis methods and population heterogeneity have posed challenges in the validation of distinct tear mediator profiles for clinical use in uveitis.^14 15 32–35^ Accordingly, tears represent a biologically plausible source of biomarkers, but a systematic synthesis of available data is required to reconcile potential differences across assay platforms, patient populations, disease definitions and treatment status.

Uveitis is a clinically heterogeneous and immunologically complex disease. Investigating the immune landscape of the cornea and tear film as a potential proxy for intraocular inflammation offers the potential to identify indicators of subclinical inflammation in anterior uveitis. Defining the clinical relevance of corneal immunological features and tear mediator profiles could support improved diagnosis, prognosis and therapy. For example, distinct corneal immune cell features may have utility for predicting an individual’s risk of relapse to inform personalised management plans. Similarly, comparison of pre-treatment and post-treatment tear mediator profiles may provide insight into treatment responsiveness to improve the precision of disease monitoring and therapeutic decision-making.

## Objectives

The primary objective of this systematic review and meta-analysis is to identify and characterise the immunological features of the human cornea and tear film in eyes with anterior uveitis (active or inactive) and healthy (control) eyes.

The secondary objectives are:

1. To examine if there are differences in corneal immunological features and tear mediator levels for different subtypes of anterior uveitis during active disease.
2. To determine the association between anterior chamber inflammation and corneal immunological features in anterior uveitis.
3. To assess for differences in corneal immunological features and tear mediator levels pre-treatment and post-treatment of anterior uveitis.

Consistent with the literature, particularly the SUN Working Group,^8^ anterior chamber cell counts of >0.5+ are considered ‘active’ and ≤0.5+ are considered ‘inactive’ anterior uveitis. If possible, data for inactive anterior uveitis eyes will be further subcategorised, and analysed, based on ‘remission’ (defined as inactive disease for at least 3 months after discontinuing all treatments for eye disease) and ‘quiescent’ (defined as inactivity while on treatment for eye disease) subgroups.

## Methods and analysis

This protocol was prepared according to the Preferred Reporting Items for Systematic review and Meta-Analysis Protocols (PRISMA-P) 2015 statement (online supplemental material 1) and prospectively registered on the International Prospective Register of Systematic Reviews (PROSPERO) on 13 April 2026 (CRD420261339682). This proposed systematic review and meta-analysis will be conducted according to the predefined protocol and reported in accordance with the Preferred Reporting Items for Systematic reviews and Meta-Analyses (PRISMA) 2020 statement.

### Eligibility criteria

Studies will be eligible for inclusion in the review if they fulfil the following eligibility criteria, described in accordance with the Participants/Patients, Interventions/Exposures, Comparator and Outcomes (PICO) framework:^36^

#### Participants

Studies need to report an anterior uveitis diagnosis in at least one human participant, or have evaluated tear samples and/or corneal tissues obtained from human participants that were diagnosed with anterior uveitis, with or without a control eye or control group. Within the studies, the anterior uveitis could be either in its active or inactive state, with no exclusion based on the anterior uveitis subtype, treatment status or disease duration. Cases of keratouveitis with associated anterior uveitis, for example varicella zoster virus, will be included. Participants of any demographic and from any setting or context will be included.

If the anatomical classification and the aetiological subtype of uveitis cannot be clearly determined from the information provided in the publication, the data will be excluded from the review. Data will also be excluded when study participants have intermediate uveitis, posterior uveitis or panuveitis, with anterior eye involvement. Data will be excluded in cases of comorbid corneal disease outside of keratouveitis, including corneal infection and inflammation not associated with the primary cause of the uveitis. When confounding factors are present that could significantly affect ocular surface immunology (e.g., comorbidities that are known to affect corneal immunology, such as human immunodeficiency virus (HIV) infection^37^) and findings from these participants are unable to be disambiguated from cases without non-comorbid confounding conditions, data will also be excluded.

#### Interventions/exposures

For the primary objective, the relevant exposure is anterior uveitis. Eligible studies will need to report identifying participants with anterior uveitis using an established clinical framework, guideline or diagnostic criteria. Studies in which anterior uveitis was indicated by self-report without a medical diagnosis will be excluded. For the secondary objective of evaluating treatment effects, all intervention or exposures relevant to the treatment of anterior uveitis will be included, with no exclusions based on type or duration of therapy.

#### Comparator

For primary comparisons, included studies will preferably have a healthy control group with no prior diagnosis of anterior uveitis, however studies without a control group will not be excluded. In studies that use the fellow eye of a unilateral anterior uveitis case as the comparator, the fellow eye will be assessed for any prior diagnosis of anterior uveitis from the publication. If there is no prior diagnosis, it will be considered a comparator. However, if it is unclear whether fellow-eye controls have a prior diagnosis of anterior uveitis, the control eyes will be excluded from the analysis.

If a study uses fellow-eye controls with inactive anterior uveitis, and reports these data separately from true fellow-eye controls (with no prior diagnosis of anterior uveitis), where possible, the inactive eyes will be further subcategorised into remission and/or quiescent subgroups.

Control data will only be included when reported in studies that include eyes with active or inactive anterior uveitis as a separate study group. If possible, findings for true healthy control eyes will be compared with fellow-eye controls without a prior diagnosis of anterior uveitis.

For the secondary objective of evaluating treatment effects, the comparator is the pre-treatment value from the same eye/participant, as reported by the study authors.

#### Study design

Full-text publications of primary research studies involving human participants or human samples/tissues of all study designs will be included. Eligible study designs include, but are not limited to, randomised controlled trials (RCTs), quasi-randomised trials, non-randomised intervention studies, prospective and retrospective cohort studies, case–control studies, cross-sectional studies and pre–post studies.

Unpublished data and non-peer-reviewed sources (e.g., conference abstracts, narrative reviews, systematic reviews, letters, case series, case reports and editorials) will be excluded. Preclinical, animal, and non-human laboratory-based studies will also be excluded.

### Search strategies

Comprehensive search strategies were developed, based on the inclusion of relevant keywords and controlled vocabulary to address the review objectives, encompassing anterior uveitis and the immunology of the human cornea and tear film. Electronic searches will be run in the following databases: Ovid MEDLINE, Ovid Embase and the Cochrane Library (Cochrane Central Register of Controlled Trials (CENTRAL)). Full search strategies, for the three databases, are provided in online supplemental material 2. Each database will be searched from inception to the date of the search. In addition, the reference lists of eligible studies will be manually screened to ensure the capture of all relevant studies. The review will only include studies published in English.

### Outcome measures

A table summarising the review objectives, exposures, comparators, outcome measures and planned analytical approaches is provided in online supplemental material 3.

#### Primary outcomes

Difference between control (healthy) eyes and eyes with (a) active anterior uveitis or (b) inactive anterior uveitis (including those in remission or quiescent, if reported), for:

1. Corneal immune cell densities (or counts, if density data are not reported), stratified by tissue layer, for:
  - Epithelium: total immune cells, putative T cells, putative dendritic cells.
  - Stroma: macrophages.
  - Endothelium: keratic precipitates (counts of whole aggregates).
2. Tear cytokine and chemokine levels.

#### Secondary outcomes

1. Difference between control (healthy) eyes and eyes with (a) active anterior uveitis or (b) inactive anterior uveitis (including those in remission or quiescent, if reported), for corneal immune cell morphology, stratified by tissue layer (epithelium, stroma, endothelium (KP)), for:
  - Quantitative characteristics, including, but not limited to, cell size, field size, field area, circularity and solidity.
  - Qualitative characteristics, including, but not limited to, cell reflectivity, shape descriptors (e.g., dendriform vs non-dendriform for KP) and pigmentation.
2. Difference between control (healthy) eyes and eyes with (a) active anterior uveitis or (b) inactive anterior uveitis (including those in remission or quiescent, if reported), for tear levels of non-cytokine and non-chemokine immune mediators, including, but not limited, to growth factors, neuropeptides, immunoglobulins, matrix metalloproteinases, arachidonic acid metabolites and complement cascade components.
3. Difference in corneal immunological features for different subtypes of anterior uveitis (e.g., infectious vs non-infectious disease).
4. Difference in tear mediator levels for different subtypes of anterior uveitis (e.g., infectious vs non-infectious disease).
5. Difference in corneal immunological features, pre-treatment versus post-treatment for anterior uveitis.
6. Difference in tear mediator levels, pre-treatment versus post-treatment for anterior uveitis.
7. Association between anterior chamber inflammation, as measured by the study investigators and corneal immunological features in anterior uveitis.

For all outcomes involving comparisons between control eyes and eyes with active and inactive anterior uveitis, if, within a particular study, no control (eye or group) data are available, information or data relevant to the features listed for each measure will be captured for anterior uveitis (active and/or inactive) only. Tear mediators will be assigned to a single category based on their predominant classification in the immunology literature.

### Data collection and analysis

#### Screening and data extraction

Systematic searches will be conducted in each electronic database and then compiled into a single library for importing into Covidence (Veritas Health Innovation, Melbourne, Australia). Any duplicate citations will be removed. Two review authors will independently assess the titles and abstracts of citations for eligibility. Citations deemed relevant or potentially relevant to the review topic will undergo full-text eligibility evaluation; this step will also be performed independently by two review authors. Reference lists of eligible studies will be manually assessed to minimise the omission of any relevant studies. At each stage, any disagreements in eligibility assessment will be resolved by discussion, or with the assistance of a third review author, if required.

A PRISMA flow diagram will be included in the final review paper to summarise the study selection process, and to report the reason for excluding studies that reach full-text screening. Following full-text review, two review authors will extract data from each study using a standardised data extraction form to ensure consistent data collection. This will include:

1. Article details: publication year, journal of publication, corresponding author details and whether the study investigators were contacted for further information.
2. Study details: type of research question (e.g., intervention, diagnostic-test accuracy, aetiology, prognosis, screening intervention), study design, setting, country conducted, date/s conducted, duration (including length of follow-up, if applicable).
3. Population details: participant eligibility criteria, number of samples (participants or eyes) per group/sample size, participant characteristics at baseline (age, sex and/or gender, ethnicity), details relating to anterior uveitis (aetiology, clinical course), anterior uveitis diagnostic criteria/definition (if reported). Where stated, we will extract information on whether a contralateral unaffected eye was used as a control versus a separate healthy control eye, to potentially consider contralateral eye effects in the analyses.
4. Treatment (intervention) details: treatment name (and comparator name, if relevant), dose, treatment duration, compliance measures, time interval between diagnosis and initiation of treatment.
5. Outcomes:
  - List of outcome measures as reported by the study authors.
  - Quantitative data will be extracted for all primary and secondary outcomes to calculate effect estimates for between-group and pre-treatment/post-treatment comparisons. For continuous outcomes, extracted data will include means and measures of variability (e.g., standard deviations (SDs), standard errors (SEs), confidence intervals (CIs)), change-from-baseline values where reported, and other summary statistics required to derive these measures. For dichotomous outcomes, effect estimates (e.g., odds ratios (ORs), risk ratios (RRs)) and corresponding measures of statistical uncertainty, or raw event data and sample sizes, will be extracted. Where necessary, alternative summary statistics will be converted to a common metric to facilitate quantitative synthesis.
6. Methodological details (as related to the outcome measures for this review): unit of analysis (per-person, per eye, average of both eyes, if applicable), investigative tool/s used, time points measured (if longitudinal).
7. Other details: source of funding statement (dichotomous: present or absent), actual source of funding (e.g., industry or government funding), conflict of interest statement (dichotomous: present or absent), conflict of interest type (e.g., employee of company conducting study).

If data are missing or further information is required to clarify if data should be included in the review, we will attempt to contact the original study investigators. If we do not receive a response from the corresponding author within 4 weeks, or if the authors are unable to provide the requested information, the information available in the publication will be used.

### Assessment of risk of bias

For each included study, risk of bias will be assessed independently by two review authors using a tool appropriate for the study design, as follows:

- The Cochrane Risk of Bias 2 tool for RCTs, according to Chapter 8 of the Cochrane Handbook of Systematic Reviews of Interventions.^38^ The risk of bias in each domain will be judged as low risk of bias, some concerns or high risk of bias.
- The Risk Of Bias In Non-randomised Studies—of Interventions, V.2 for non-randomised intervention studies, controlled interrupted-time series and controlled before–after studies.^39^ The risk of bias in each domain will be judged as low, moderate, serious or critical.
- The Risk Of Bias In Non-randomised Studies—of Exposures, for observational cohort studies, case–control studies and observational epidemiological studies with exposure assessment.^40^ The risk of bias in each domain will be judged as low risk of bias, some concerns, high risk of bias or very high risk of bias.
- The National Institutes of Health (NIH) Quality Assessment Tools for all other study designs, including pre–post studies with no control group, uncontrolled interrupted time-series, non-interventional cross-sectional studies and case series. Risk of bias will be assessed according to the design-specific criteria of the relevant NIH assessment tool, with each criterion judged as yes, no, or other (cannot determine, not reported or not applicable), and an overall quality rating (good, fair, or poor) determined by level of bias.^41^

Any disparities in assessment between review authors will be resolved via discussion to reach consensus, or consulting a third review author (if/when required). Risk of bias assessments will be summarised using tables and/or figures.

#### Assessment of publication biases

Where 10 or more studies are included in a meta-analysis, potential reporting biases will be assessed by visualising asymmetry in a funnel plot and small-study effects will be considered using Egger’s test.

### Data synthesis

#### Primary outcomes

For the difference in corneal immune cell densities, and tear cytokine and chemokine levels, between healthy eyes (without a prior diagnosis of anterior uveitis) and eyes with (a) active anterior uveitis or (b) inactive anterior uveitis (including those in remission or quiescent, if reported), a meta-analysis will be performed (for each comparison) when two or more studies report quantitative data for the same pre-specified outcome measure. As all primary outcome measures are continuous, mean differences (MDs) will be used when studies report the outcome measure in the same units, and standardised mean differences when units differ.

Data will be synthesised across studies using inverse-variance weighted meta-analyses with 95% CIs. A random-effects model will be applied when more than three studies contribute data, and a fixed-effect model when three or fewer studies are pooled. Statistical heterogeneity will be quantified using the I^²^ statistic, with I^²^ values ≥60% considered to indicate significant statistical heterogeneity.^42 43^

#### Secondary outcomes

For the difference in corneal immune cell morphology and various tear mediator levels between healthy eyes and eyes with (a) active anterior uveitis, or (b) inactive anterior uveitis (including those in remission or quiescent, if reported), a meta-analysis will be performed when two or more studies report quantitative data for the same prespecified outcome measure. Qualitative characteristics of corneal immune cell morphology (e.g., cell reflectivity, shape descriptors, pigmentation) will be grouped into predefined categories and synthesised narratively.

To evaluate the relationship between anterior chamber inflammation and corneal immunological features in anterior uveitis, a meta-analysis will be performed if more than one study reports relevant quantitative data suitable for comparison. Studies will be grouped by anterior chamber inflammation status, dichotomised into active inflammation (SUN grade >0.5+) and inactive inflammation (SUN grade ≤0.5+). Studies will be included in the meta-analysis only if anterior chamber inflammation is reported using the SUN criteria for anterior chamber cells,^8^ or if alternative grading systems can be converted to equivalent SUN grades. If the grading method is unclear or cannot be reliably converted to the SUN scale, the study will be excluded from the quantitative synthesis of this outcome.

To consider the effect of treatment on the immunological features of the cornea and/or tear film in anterior uveitis in the absence of a control group, a meta-analysis will be performed if more than one study reports quantitative pre-treatment versus post-treatment data on the same prespecified review outcome measure at comparable time points. Information on the change in mean from baseline, and SD of change, at pre-treatment and post-treatment time points, will be extracted. If change data are not reported, information on the mean and SD of the outcome, at pre-treatment and post-treatment time points will be extracted with estimates of correlation between pre-treatment and post-treatment if available. The effects of the interventions will be expressed as the MD, with 95% CIs, between time points.

For primary and secondary outcomes where a meta-analysis is not possible due to insufficient data or significant heterogeneity, a narrative synthesis will be provided.

### Sensitivity analysis

A sensitivity analysis will be performed for the primary outcome measures if sufficient data are available. This analysis will assess the impact of excluding studies that were judged to have a high risk of bias overall.

### Subgroup analysis

The following subgroup analyses are directly relevant to addressing the secondary objectives of the review, as related to:

- Whether different subtypes of anterior uveitis show distinct corneal and/or tear film immunological features. Subgroup analyses will be conducted if three or more studies report quantitative data on the same uveitis aetiological subtype, for the same outcome measure.
- The effect of treatment on corneal and/or tear film immunological features. Subgroup analyses by potential intervention effect modifiers (e.g., type of therapeutic agent) will be performed if three or more studies report quantitative data on the same treatment type for the same outcome measure.

For all other outcomes, subgroup analyses will be conducted for parameters where there are three or more studies reporting data of a particular parameter in each subgroup category, as related to:

- Participant age group (children vs adults).
- Tool/device used to quantify the parameter.
- Tissue location, including eccentricity.
- Contralateral eye effects (i.e., contralateral unaffected eyes vs true (separate) control eyes).

## Discussion

In undertaking this systematic review, we aim to identify, appraise and synthesise available evidence relating to the immunological features of anterior uveitis in the human cornea and tear film. Understanding the effects of anterior uveitis on these ocular surface components may provide a basis for leveraging features of the cornea and/or tear film as diagnostic or prognostic indicators of the disease.

The search for novel biomarkers of disease status in anterior uveitis recognises that current approaches have limitations. At present, treatment cessation is typically guided by a clinician observing an absence of anterior chamber cellular activity (i.e., SUN grade ≤0.5+).^44^ However, a substantial proportion of uveitis cases relapse despite apparent clinical quiescence,^45^ suggesting that immune activity may persist beyond what is detectable using conventional clinical techniques. Furthermore, a first acute anterior uveitis event may subsequently have a recurrent course, and although certain risk factors have been identified (e.g., HLA-B27 positivity),^46^ there remains a lack of validated biomarkers or predictive features to reliably forecast an individual’s risk of recurrence.^47 48^ This prognostic uncertainty contributes to patient burden; for example, Prem Senthil *et al* reported that patients have trouble coping with the unpredictability of uveitis relapses.^49^

Key strengths of this protocol are the adoption of robust search strategies in multiple electronic databases, with a comprehensive strategy for each PICO element without restrictions on study design. Prospective registration on the public PROSPERO registry ensures a transparent and reproducible review process. Having two review authors engaged at each stage of the review process minimises the potential for bias and/or human error. Furthermore, adherence to established systematic review methodological standards (e.g., the Cochrane Handbook), combined with the planned use of both statistical and qualitative synthesis methods will enable analysis of diverse data types, to the extent this is possible.

A possible limitation is the exclusion of grey literature and unpublished works. This is a conscious decision to minimise the inclusion of non-peer-reviewed evidence; however, it is acknowledged that this may increase the risk of publication bias. Restricting studies to those published in English may also introduce language bias. Another challenge may be the heterogeneity of the data, particularly stemming from different investigative techniques used to analyse the human cornea and tear film. Finally, given the absence of a universally agreed diagnostic approach for anterior uveitis,^47^ study-specific classification systems will be accepted provided the criteria are clearly documented and justified by the authors. However, variable diagnostic approaches and/or disease definitions across studies may limit the consistency of synthesised results.

This planned systematic review and meta-analysis will comprehensively evaluate the available evidence on human corneal and tear film immunological features in anterior uveitis. While the review may identify candidate immunological features associated with anterior uveitis, further validation in well-designed prospective studies will be required before any identified features could be considered clinically useful biomarkers. Nevertheless, improving our understanding of ocular surface immunological features may provide novel insight into anterior segment pathology in anterior uveitis, and lay the groundwork for future research aimed at improving diagnosis, monitoring and therapy in anterior uveitis.

### Ethics and dissemination

Ethics approval is not applicable for this systematic review as no original data will be collected. The results of this review are expected to be disseminated through a peer-reviewed publication and/or scientific conference presentations.

## Supplementary material

10.1136/bmjopen-2026-121276 online supplemental file 1

10.1136/bmjopen-2026-121276 online supplemental file 2

10.1136/bmjopen-2026-121276 online supplemental file 3
