# Supplementary material for: Corneal and tear film immunology in anterior uveitis: protocol for a systematic review and meta-analysis
Source: BMJ Open. 2026 Aug 12;16(8):e121276. doi: 10.1136/bmjopen-2026-121276 (PMC13475445; doi:10.1136/bmjopen-2026-121276)
Supplement: Supplementary file 1 [file bmjopen-2026-121276.reviewer_comments.pdf]

PEER REVIEW HISTORY

BMJ Open publishes all reviews undertaken for accepted manuscripts. Reviewers are asked to complete a checklist review form and are provided with free text boxes to elaborate on their assessment. These free text comments are reproduced below.

ARTICLE DETAILS

Title (Provisional)

Corneal and tear film immunology in anterior uveitis: protocol for a systematic review and meta-analysis

Authors

Ye, Jessie L.; Tong, Janelle; Wu, Ching Yi; Fraser, Alexander S.; McGuinness, Myra; Lim, Lyndell; Downie, Laura Elizabeth

---

VERSION 1 - REVIEW

**Reviewer**	**1**
**Name**	**Nouraeinejad, Ali**
**Affiliation**	**Department of Clinical Ophthalmology, University College London**
**Date**	**13-May-2026**
**COI**	**None**

Thank you for looking into anterior uveitis. My comments are as follows:

1. It is important to apply internationally recognized terms in uveitis. The term “historical” is an odd term used in the inflammatory content of uveitis throughout the manuscript. Instead, resolved, quiescent, or inactive is suggested for use.

2. The time interval between diagnosis and the start of treatment options in patients with anterior uveitis can also be important, as this affects the results observed.

**Reviewer**	**2**
**Name**	**Ho, Kam Chun**
**Affiliation**	**University of Canberra, Discipline of Optometry and Vision Science**
**Date**	**24-May-2026**
**COI**	**None**

Thank you for the opportunity to review this protocol. This is a clinically relevant and well-developed protocol for a systematic review and meta-analysis on corneal and tear film immunological features in anterior uveitis. The rationale is generally sound, and the protocol appears broadly consistent with the PROSPERO registration. The manuscript would benefit from several clarifications to improve readability, reproducibility, and alignment between the review objectives, eligibility criteria, outcomes, and analysis plan.

1. Introduction and rationale

Pages 3-6

The Introduction provides relevant and well-informed background, but the linkage between paragraphs could be strengthened so that the reader can more easily follow how each paragraph leads to the review objective. At present, the Introduction moves from the clinical burden, classification and aetiological complexity of anterior uveitis, to limitations of current clinical assessment, then to keratic precipitates, corneal immune cells, imaging methods and tear film mediators. These topics are all relevant, but the connection to the central review focus, namely corneal and tear film immunology in anterior uveitis, is not always made explicit until the final rationale paragraph and Objectives section.

Please consider adding brief transitional or concluding sentences to key paragraphs to clarify how each section supports the rationale for systematically reviewing corneal immune features and tear film mediators. In particular, the paragraph on current clinical assessment could more clearly lead into the need for complementary biomarkers of ocular immune activity; the paragraphs on keratic precipitates and corneal immune cells could more explicitly frame these as corneal immunological features relevant to the review; and the tear film paragraph could more clearly highlight the need to synthesise heterogeneous evidence across assay methods, populations, disease definitions and treatment states.

The Introduction could also better prepare the reader for the secondary objective relating to pre- versus post-treatment changes. Although treatment responsiveness is mentioned in the rationale, the Introduction does not clearly explain why changes in corneal immunological features or tear mediator levels after treatment are important to this review. Please consider adding a brief link between treatment-related changes and the potential role of these features as markers of disease activity or treatment responsiveness.

2. Objectives

Page 6

The secondary objective refers to determining the “correlation” between anterior chamber inflammation severity and corneal immunological features. This is appropriate if the intended analysis is limited to studies reporting correlation coefficients. However, relevant studies may report this relationship in other ways, such as comparisons of corneal immune features across SUN grades or other inflammation severity categories. Please consider whether the broader term “association” would better reflect the intended objective, while

retaining correlation coefficients in the data synthesis section for studies that report suitable quantitative correlation data.

3. Eligibility criteria

Pages 6-8

In the Participants section, please clarify the inclusion of studies involving “human tissues”. The current wording refers to human participants or human tissues with anterior uveitis. Because anterior uveitis is a clinical diagnosis rather than a property of tissue alone, please clarify whether tissue-only or sample-based studies are eligible only when the tissue or sample is obtained from human eyes or participants with clinically diagnosed active or historical anterior uveitis.

In the Interventions/Exposures section, the relevant exposure for the main review question appears to be anterior uveitis status, namely active anterior uveitis or historical anterior uveitis, rather than treatment. The current section mainly discusses diagnostic confirmation of anterior uveitis and then treatment exposures relevant to pre-/post-treatment analyses. Please clarify this section by distinguishing anterior uveitis status as the exposure or condition of interest for the main analyses from treatment exposures, which appear relevant specifically to the secondary objective assessing pre- versus post-treatment changes.

In the Comparator section, healthy controls and fellow-eye controls are described clearly for the primary comparison. However, the protocol also includes pre- versus post-treatment outcomes. Please clarify the comparator for these treatment-related analyses. Presumably, the comparator is the pre-treatment value from the same eye or participant, where available.

4. Search strategy

Page 8 and Supplementary Material 2

Please clarify the Search Strategies section by briefly describing and justifying the conceptual blocks used to develop the search terms. While the detailed search strategy can remain in the supplementary material, the main text should make clear how the search terms were formed and how the search structure aligns with the review objectives.

5. Outcome definitions

Pages 9-10

The outcomes are broadly appropriate and aligned with the review objective, but several definitions should be clarified.

First, please clarify the distinction between “tear cytokine levels” as a primary outcome and broader “tear mediator levels” as a secondary outcome. Many immunoassay panels report cytokines, chemokines, growth factors and other inflammatory proteins together. It would therefore be helpful to specify how mediator classes will be categorised and how overlapping categories will be handled.

Second, please clarify how keratic precipitates will be handled within the corneal immune cell density outcome. Keratic precipitates may be reported as density, counts, morphology, distribution or qualitative descriptors rather than directly as immune cell density. Please clarify whether KP density/counts are primary outcomes and whether KP morphology/distribution will be treated as secondary outcomes.

Third, as noted above, the outcome relating anterior chamber inflammation severity to corneal immunological features may be better framed as an association rather than only a correlation, unless the authors intend to restrict this outcome to studies reporting correlation coefficients.

6. Risk-of-bias assessment

Pages 11-12

The risk-of-bias assessment section is generally clear and consistent with the PROSPERO record, but please justify the choice of tools for the range of study designs expected in this review. In particular, ROBINS-I V2 is proposed for observational cohort, case-control and cross-sectional studies, but the review primarily concerns corneal and tear film immunological features rather than only intervention effects. Please clarify whether ROBINS-I V2 is appropriate for these observational biomarker or association studies and, if so, briefly explain how it will be applied. If different tools will be used according to study question or design, please specify this.

Please also revise the RoB 2 judgement categories. The manuscript states “low, medium, or high risk”, whereas standard RoB 2 terminology is “low risk of bias”, “some concerns”, and “high risk of bias”.

7. Data synthesis

Pages 12-13

The Data Synthesis section is generally clear, but several aspects of the planned synthesis require clarification.

First, please justify the decision to use a fixed-effect model when three or fewer studies are pooled and a random-effects model when more than three studies are pooled. Given the likely clinical and methodological heterogeneity across anterior uveitis subtypes, tissue layers, imaging methods, assay platforms, treatment status and outcome definitions, model choice may need to be guided by expected heterogeneity rather than only the number of studies.

Second, please specify the planned effect measures for the different categories of secondary outcomes. These include group comparisons, qualitative morphology, subtype comparisons, pre-/post-treatment changes and associations with anterior chamber inflammation severity.

Third, for pre-/post-treatment analyses, please clarify how paired eye-level or participant-level data will be handled, including how missing SDs of change or within-person/within-eye correlations will be addressed.

Finally, for the inflammation severity outcome, please clarify whether studies reporting categorical comparisons across inflammation grades, rather than correlation coefficients, will be included and how they will be synthesised.

8. Sensitivity and subgroup analyses

Page 14

The planned sensitivity and subgroup analyses are generally appropriate. Please clarify whether the sensitivity analysis excluding studies at high risk of bias will be based on overall risk of bias only, or on specific domains such as participant selection.

The Subgroup Analysis section lists several possible subgroup variables, including uveitis subtype, treatment type, participant age group, measurement tool/device, tissue location and contralateral-eye effects. Please clarify which subgroup analyses are considered a priori and most directly linked to the review objectives, and how subgroup findings will be interpreted given the likely small number of studies and expected methodological heterogeneity.

9. Suggested summary table

Methods section (overall)

Given the number of review questions, outcomes, comparators and planned analyses, the Methods section would be easier to follow if the authors included a summary table linking each review objective/question to the relevant exposure or comparison, comparator group, outcomes of interest, outcome measures, planned synthesis method, and relevant subgroup analyses. At present, the protocol includes several related but distinct questions, including active or historical anterior uveitis versus healthy controls, differences between anterior uveitis subtypes, associations with anterior chamber inflammation severity, and pre- versus post-treatment changes. These questions involve different comparators and different types of analysis, which can be difficult to follow when presented only in text. A summary table would improve clarity, reproducibility, and alignment between the objectives, outcomes and analysis plan.

10. Discussion and limitations

Pages 14-16

The Discussion is generally appropriate for a protocol and clearly explains the potential importance of synthesising evidence on corneal and tear film immunological features in anterior uveitis. However, the limitations section could be strengthened.

In addition to the stated limitations around exclusion of grey literature, heterogeneity of investigative techniques, and variability in diagnostic definitions, please acknowledge the

English-language restriction as a potential source of language bias. Please also balance the statement about excluding grey literature: while this may reduce reliance on non-peer-reviewed sources, it may also increase the risk of publication bias.

Finally, please ensure that statements about the clinical potential of corneal or tear film biomarkers remain appropriately cautious. This review can identify and synthesise candidate immunological features, but further validation would be needed before these features could be considered clinically useful biomarkers.

VERSION 1 - AUTHOR RESPONSE

Reviewer 1 Comments	Authors' Response
1. It is important to apply internationally recognized terms in uveitis. The term "historical" is an odd term used in the inflammatory content of uveitis throughout the manuscript. Instead, resolved, quiescent, or inactive is suggested for use.	Further to the reviewer's suggestion, we have revised this terminology throughout the manuscript (pages 6, 7, 8, 10, 11, and 14): References to 'historical' anterior uveitis have been replaced with 'inactive' anterior uveitis. Consideration has also been given to sub-categorise inactive anterior uveitis into 'remission' and 'quiescent' subgroups, in accordance with terminology used in the literature (Jabs et al., 2005; Suhler et al., 2018). In addition, the phrase 'history of uveitis' has been replaced with 'no prior diagnosis of uveitis' when referring to a person's ophthalmic history.
2. The time interval between diagnosis and the start of treatment options in patients with anterior uveitis can also be important, as this affects the results observed.	We agree that the time interval between diagnosis and the initiation of treatment may influence outcomes. Accordingly, we have added this variable to the data extraction template.<br>• **Page 12, Data Extraction – Treatment (intervention) details:** "Treatment (intervention) details: treatment name (and comparator name, if relevant), dose, treatment duration, compliance measures, time interval between diagnosis and initiation of treatment."

Reviewer 2 Comments	Authors' Response
1. Introduction and rationale Pages 3-6; The Introduction provides relevant and well-informed	We thank the reviewer for this helpful comment. We acknowledge that the connections between the background

background, but the linkage between paragraphs could be strengthened so that the reader can more easily follow how each paragraph leads to the review objective. At present, the Introduction moves from the clinical burden, classification and aetiological complexity of anterior uveitis, to limitations of current clinical assessment, then to keratic precipitates, corneal immune cells, imaging methods and tear film mediators. These topics are all relevant, but the connection to the central review focus, namely corneal and tear film immunology in anterior uveitis, is not always made explicit until the final rationale paragraph and Objectives section.<br><br>Please consider adding brief transitional or concluding sentences to key paragraphs to clarify how each section supports the rationale for systematically reviewing corneal immune features and tear film mediators. In particular, the paragraph on current clinical assessment could more clearly lead into the need for complementary biomarkers of ocular immune activity; the paragraphs on keratic precipitates and corneal immune cells could more explicitly frame these as corneal immunological features relevant to the review; and the tear film paragraph could more clearly highlight the need to synthesise heterogeneous evidence across assay methods, populations, disease definitions and treatment states.<br><br>The Introduction could also better prepare the reader for the secondary objective relating to pre- versus post-treatment changes.	sections and the review objectives could be made more explicit. We have revised the Introduction (pages 3-6) accordingly.<br><br>Specifically, we added a transitional statement following the consideration of current clinical assessment to "highlight the potential benefit of complementary, objective markers that reflect underlying ocular immune activity as a means to improve disease characterisation, monitoring and prognostication in anterior uveitis."<br><br>We have added a concluding sentence after the section on keratic precipitates and corneal immune cells, to explicitly frame these as key corneal immunological features relevant to the review: "It is thus timely to undertake the present review, involving the collation and synthesis of evidence relating to how corneal immunology might be altered in anterior uveitis, and in response to treatment, as a foundation for considering how these features could serve as meaningful indicators of ocular inflammation and/or disease quiescence."<br><br>We revised the tear film section to explicitly acknowledge the heterogeneity in assay platforms, patient populations, disease definitions, and treatment status across studies, thereby reinforcing the need for systematic synthesis of the available evidence: "Accordingly, tears represent a biologically plausible source of biomarkers, but systematic synthesis of available data is required to reconcile potential differences across assay platforms, patient populations, disease definitions, and treatment status."<br><br>To better support the secondary objective, we have added text explaining the relevance of treatment-related changes in corneal immunological features and tear mediator profiles: "…comparison of pre- and post-treatment ~~a~~ tear mediator profiles may ~~indicative~~ provide insight into treatment responsiveness, ~~of sub-clinical inflammation,~~ to improve the precision of disease monitoring and therapeutic decision making-

<table>
<tr>
<td>Although treatment responsiveness is mentioned in the rationale, the Introduction does not clearly explain why changes in corneal immunological features or tear mediator levels after treatment are important to this review. Please consider adding a brief link between treatment-related changes and the potential role of these features as markers of disease activity or treatment responsiveness.</td>
<td>~~may increase the precision of therapy~~.” The Introduction now highlights that evaluating pre- and post-treatment changes in corneal and tear film immunological features may provide insight into disease activity/quiescence, treatment responsiveness, and subclinical inflammation, as a foundation for considering whether such measures may be potentially useful biomarkers in anterior uveitis.</td>
</tr>
<tr>
<td>2. Objectives Page 6;
The secondary objective refers to determining the “correlation” between anterior chamber inflammation severity and corneal immunological features. This is appropriate if the intended analysis is limited to studies reporting correlation coefficients. However, relevant studies may report this relationship in other ways, such as comparisons of corneal immune features across SUN grades or other inflammation severity categories. Please consider whether the broader term “association” would better reflect the intended objective, while retaining correlation coefficients in the data synthesis section for studies that report suitable quantitative correlation data.</td>
<td>We thank the reviewer for this suggestion and agree that ‘association’ is a more appropriate term than ‘correlation’ for this objective. We have revised the review objective, and corresponding secondary outcome, to accommodate studies reporting relationships between anterior chamber inflammation severity and corneal immunological features using a range of analytical approaches.
• **Page 6, Objectives:** “To determine the ~~correlation~~ association between ~~the severity of~~ anterior chamber inflammation ~~in active anterior uveitis~~ and corneal immunological features in anterior uveitis.”
• **Page 10, Secondary Outcomes:** ~~Correlation “~~Association between ~~the severity of~~ anterior chamber inflammation,~~ in active anterior uveitis~~, as measured by the study investigators, and corneal immunological features in anterior uveitis.”</td>
</tr>
<tr>
<td>3. Eligibility criteria Pages 6-8;
In the Participants section, please clarify the inclusion of studies involving “human tissues”. The current wording refers to human participants or human tissues with anterior uveitis. Because anterior uveitis is a clinical diagnosis rather than a property of tissue alone, please clarify whether tissue-only or sample-based studies are eligible only when the tissue or</td>
<td>Further to the reviewer’s comment, we have clarified in the manuscript that tissue- or sample-based studies are only eligible where specimens are obtained from human participants with clinically diagnosed anterior uveitis.
• **Page 7, Participants:** “Studies need to report an anterior uveitis diagnosis in at least one human participant, or have evaluated tear samples and/or corneal tissues obtained from human</td>
</tr>
</table>

sample is obtained from human eyes or participants with clinically diagnosed active or historical anterior uveitis.<br><br>In the Interventions/Exposures section, the relevant exposure for the main review question appears to be anterior uveitis status, namely active anterior uveitis or historical anterior uveitis, rather than treatment. The current section mainly discusses diagnostic confirmation of anterior uveitis and then treatment exposures relevant to pre-/post-treatment analyses. Please clarify this section by distinguishing anterior uveitis status as the exposure or condition of interest for the main analyses from treatment exposures, which appear relevant specifically to the secondary objective assessing pre- versus post-treatment changes.<br><br>In the Comparator section, healthy controls and fellow-eye controls are described clearly for the primary comparison. However, the protocol also includes pre- versus post-treatment outcomes. Please clarify the comparator for these treatment-related analyses. Presumably, the comparator is the pre-treatment value from the same eye or participant, where available.	participants that were diagnosed with anterior uveitis…"<br><br>We have distinguished anterior uveitis (active or inactive) as the primary exposure of interest from treatment-related exposures, which are relevant only to secondary pre- versus post-treatment analyses.<br>• **Page 8, Interventions/Exposures:** "For the primary objective, the relevant exposure is anterior uveitis. Eligible studies will need to report identifying participants with anterior uveitis using an established clinical framework, guideline or diagnostic criteria. Studies in which anterior uveitis was indicated by self-report without a medical diagnosis will be excluded. For the secondary objective of evaluating treatment effects, all intervention or exposures relevant to the treatment of anterior uveitis will be included, with no exclusions based on type or duration of therapy."<br><br>In addition, we have clarified that for the secondary outcome of treatment-related analyses, the comparator is the pre-treatment value from the same eye or participant, where available<br>• **Page 9, Comparator:** "For the secondary objective of evaluating treatment effects, the comparator is the pre-treatment value from the same eye/participant, as reported by the study authors."
4. Search strategy Page 8 and Supplementary Material 2;<br>Please clarify the Search Strategies section by briefly describing and justifying the conceptual blocks used to develop the search terms. While the detailed search strategy can remain in the supplementary material, the main text should make clear how the search terms were formed and how the search	We have clarified the conceptual rationale for the construction of the electronic search strategies in the manuscript, and included the full search strategy for each electronic database in supplementary material.<br>• **Page 9, Search Strategies:** "Comprehensive search strategies ~~to identify all relevant studies will be undertaken in~~ were developed, based on the inclusion of relevant keywords and controlled vocabulary to address the review objectives, encompassing

structure aligns with the review objectives.	anterior uveitis and the immunology of the human cornea and tear film. Electronic searches will be run in the following databases: Ovid MEDLINE, Ovid Embase and the Cochrane Library (Cochrane Central Register of Controlled Trials [CENTRAL]). ~~A representative~~ Full search strategies, for the three databases, are provided in Supplementary Material 2.”
5. Outcome definitions Pages 9-10; The outcomes are broadly appropriate and aligned with the review objective, but several definitions should be clarified.<br><br>First, please clarify the distinction between “tear cytokine levels” as a primary outcome and broader “tear mediator levels” as a secondary outcome. Many immunoassay panels report cytokines, chemokines, growth factors and other inflammatory proteins together. It would therefore be helpful to specify how mediator classes will be categorised and how overlapping categories will be handled.<br><br>Second, please clarify how keratic precipitates will be handled within the corneal immune cell density outcome. Keratic precipitates may be reported as density, counts, morphology, distribution or qualitative descriptors rather than directly as immune cell density. Please clarify whether KP density/counts are primary outcomes and whether KP morphology/distribution will be treated as secondary outcomes.<br><br>Third, as noted above, the outcome relating anterior chamber inflammation severity to corneal immunological features may be better framed as an association	We thank the reviewer for this comment.<br><br>First, re: the potential distinction between ‘tear cytokine levels’ and ‘tear mediator levels’, our intention was for ‘mediators’ to refer to a broader category of molecules involved in immune signalling other than cytokines, but we appreciate the need for clarification for the specification of outcome measures. This has been addressed in the manuscript:<br>• **Page 10, Primary Outcomes:** “2. Tear cytokine and chemokine levels”<br><br>• **Page 10, Secondary Outcomes:** “2. … tear levels of non-cytokine and non-chemokine ~~proteins~~ immune mediators, including but not limited to growth factors, neuropeptides, immunoglobulins, matrix metalloproteinases, arachidonic acid metabolites, and complement cascade components.”<br>“Tear mediators will be assigned to a single category based on their predominant classification in the immunology literature.”<br><br>Second, re: how keratic precipitate (KP) outcomes will be handled, this has been clarified in the manuscript:<br>• **Page 10, Primary Outcomes:** “1. Corneal immune cell densities (or counts, if density data are not reported), stratified by tissue layer, for:<br>• Epithelium: total immune cells, putative T cells, putative dendritic cells;<br>• Stroma: macrophages;

rather than only a correlation, unless the authors intend to restrict this outcome to studies reporting correlation coefficients.	• Endothelium: keratic precipitates (counts of whole aggregates)."<br><br>• **Page 10, Secondary Outcomes:** "1. Difference between control (healthy) eyes and eyes with (i) active anterior uveitis or (ii) inactive anterior uveitis (including those in remission or quiescent, if reported), for corneal immune cell morphology, stratified by tissue layer (epithelium, stroma, endothelium [KP]), for:<br>• Quantitative characteristics, including, but not limited to, cell size, field size, field area, circularity, and solidity.<br>• Qualitative characteristics, including, but not limited to, cell reflectivity, shape descriptors (e.g., dendriform vs non-dendriform for KP), and pigmentation."<br><br>Third, re: per the response to comment #2, the relevant terminology has been changed from 'correlation' to 'association'.<br>• **Page 6, Objectives:** "To determine the ~~correlation~~ association between ~~the severity of~~ anterior chamber inflammation ~~in active anterior uveitis~~ and corneal immunological features in anterior uveitis."<br>• **Page 10, Secondary Outcomes:** ~~Correlation~~ "Association between ~~the severity of~~ anterior chamber inflammation, ~~in active anterior uveitis~~, as measured by the study investigators, and corneal immunological features in anterior uveitis."
6. Risk-of-bias assessment Pages 11-12; The risk-of-bias assessment section is generally clear and consistent with the PROSPERO record, but please justify the choice of tools for the range of study designs expected in this review. In particular, ROBINS-I V2 is proposed for observational	We thank the reviewer for raising this point.<br><br>We agree that ROBINS-I V2 is designed for non-randomised studies of interventions, and other risk of bias tools are more appropriate for observational studies.<br><br>We have revised the section on risk of bias in the manuscript, to ensure all potential study

cohort, case-control and cross-sectional studies, but the review primarily concerns corneal and tear film immunological features rather than only intervention effects. Please clarify whether ROBINS-I V2 is appropriate for these observational biomarker or association studies and, if so, briefly explain how it will be applied. If different tools will be used according to study question or design, please specify this.<br><br>Please also revise the RoB 2 judgement categories. The manuscript states "low, medium, or high risk", whereas standard RoB 2 terminology is "low risk of bias", "some concerns", and "high risk of bias".	designs are mapped to an appropriate risk of bias tool, and to more clearly define the risk of bias judgement categories:<br>**Page 13, Assessment of Risk of Bias:**<br>• "The Cochrane Risk of Bias 2 (RoB2) tool for RCTs, according to Chapter 8 of the Cochrane Handbook of Systematic Reviews of Interventions.[38] The risk of bias in each domain will be judged as low risk of bias, some concerns, or high risk of bias.<br>• The Risk Of Bias In Non-randomised Studies - of Interventions, Version 2 (ROBINS-I V2) for non-randomised intervention studies, ~~observational cohort studies, case-control studies, cross-sectional studies~~, controlled interrupted-time series, and controlled before-after studies.[39] The risk of bias in each domain will be judged as low, moderate, serious, or critical.<br>• The Risk Of Bias In Non-randomised Studies - of Exposures (ROBINS-E), for observational cohort studies, case-control studies, and observational epidemiological studies with exposure assessment.[40] The risk of bias in each domain will be judged as low risk of bias, some concerns, high risk of bias, or very high risk of bias.<br>• The National Institutes of Health (NIH) Quality Assessment Tools for all other study designs, including ~~for Pre-Post Studies With No Control Group, for~~ pre-post studies with no control group, uncontrolled interrupted time-series, non-interventional cross-sectional studies, and case series. Risk of bias will be assessed according to the design-specific ~~a set of 12~~ criteria of the relevant NIH assessment tool, with each criterion judged as ~~judged as~~ yes, no, or other (cannot determine, not reported, or not applicable), ~~with~~ and an overall quality rating (good, fair, or poor) determined by level of bias.[41]"
7. Data synthesis Pages 12-13; The Data Synthesis section is	We appreciate the reviewer's queries.

generally clear, but several aspects of the planned synthesis require clarification.<br><br>First, please justify the decision to use a fixed-effect model when three or fewer studies are pooled and a random-effects model when more than three studies are pooled. Given the likely clinical and methodological heterogeneity across anterior uveitis subtypes, tissue layers, imaging methods, assay platforms, treatment status and outcome definitions, model choice may need to be guided by expected heterogeneity rather than only the number of studies.<br><br>Second, please specify the planned effect measures for the different categories of secondary outcomes. These include group comparisons, qualitative morphology, subtype comparisons, pre-/post-treatment changes and associations with anterior chamber inflammation severity.<br><br>Third, for pre-/post-treatment analyses, please clarify how paired eye-level or participant-level data will be handled, including how missing SDs of change or within-person/within-eye correlations will be addressed.<br><br>Finally, for the inflammation severity outcome, please clarify whether studies reporting categorical comparisons across inflammation grades, rather than correlation coefficients, will be included and how they will be synthesised.	First, re: the specification of a fixed-effect model when three or fewer studies are pooled, this deliberate choice has been retained. Estimates of between-study variance are needed for random-effects meta-analyses and can be unreliable without sufficient studies. It has been previously noted that “random-effects meta-analyses with only a few studies often face challenges in accurately estimating between-study heterogeneity, leading to biased effect estimates and confidence intervals with poor coverage” (Duan et al., 2025). Further, the Cochrane Handbook states that “Careful interpretation of random-effects meta-analysis with few studies is required because neither the Wald-type nor the HKSJ method provides a completely satisfactory solution to the technical difficulty of estimating the between-study variance when there are few studies.”<br><br>Second, re: the planned effect measures for the secondary outcomes, we have added a table summarising the exposures, comparators, outcome measures and planned analytical approaches for each review objective (Supplementary Material 3). Considering that we intend to report on multiple outcomes and with an expectation that individual study authors may have taken different approaches to quantify and/or report relevant outcomes, we have intentionally chosen not to be prescriptive regarding how effect measures will be reported. Instead, we have described the overall analytical approach in the context of how data will be aggregated “when two or more studies report quantitative data”, for relevant comparisons, as well as for the pre-/post-treatment changes, and relationships between outcomes (e.g., anterior chamber inflammation status and corneal immunological features) (pages 14-15). We also state that “For primary and secondary outcomes where a meta-analysis is not possible due to insufficient data or significant heterogeneity, a narrative synthesis will be provided” (page 15).

	Third, re: pre-/post-treatment analyses, we will derive the standard deviation (SD) for the within-group mean change from baseline if no measure of variance is reported in the original report or provided following contact with authors. This SD will be derived using the SDs reported pre- and post-treatment and the correlation between the two time points. Correlation coefficients may be imputed based on previous literature if not provided by the original study authors.<br>**Page 15, Secondary Outcomes:**<br>• "To consider the effect of treatment on the immunological features of the cornea and/or tear film in anterior uveitis in the absence of a control group, a meta-analysis will be performed if more than one study reports quantitative pre- vs post-treatment data on the same pre-specified review outcome measure at comparable timepoints. Information on the change in mean from baseline, and standard deviation (SD) of change, at pre- and post-treatment timepoints, will be extracted. If change data are not reported, information on the mean and SD of the outcome, at pre- and post-treatment timepoints, will be extracted with estimates of correlation between pre- and post-treatment if available. The effects of the interventions will be expressed as the mean difference (MD), with 95% confidence intervals (CIs), between timepoints."<br><br>Fourth, re: the anterior chamber inflammation outcome, we have revised the secondary objective to more clearly specify the approach. We have dichotomised anterior chamber inflammation into active (SUN > 0.5+) and inactive (SUN ≤ 0.5+) groups based on the SUN criteria for anterior chamber cells. The meta-analysis will focus on studies that report quantitative data stratified by inflammation grade/status. Studies reporting only correlation coefficients between anterior chamber inflammation and corneal immunological features will be

excluded from this meta-analysis, however, will be included in the narrative summary where relevant. Please find the corresponding changes throughout the manuscript:

Page 6, Secondary Objectives:

- "To determine the association between ~~the severity of~~ anterior chamber inflammation ~~in active anterior uveitis~~ and corneal immunological features in anterior uveitis."

Page 10, Secondary Outcomes:

- "~~Correlation~~ Association between ~~the severity of~~ anterior chamber inflammation, ~~in active anterior uveitis~~ as measured by the study investigators, and corneal immunological features, in anterior uveitis."

Page 12, Data Extraction – Outcomes:

- "~~The correlation coefficient for variables of interest, including severity of anterior chamber inflammation and corneal immunological features;~~ Quantitative data will be extracted for all primary and secondary outcomes to calculate effect estimates for between-group and pre-/post-treatment comparisons. For continuous outcomes, extracted data will include means and measures of variability (e.g., standard deviations, standard errors, confidence intervals), change-from-baseline values where reported, and other summary statistics required to derive these measures. For dichotomous outcomes, effect estimates (e.g., odds ratios, risk ratios) and corresponding measures of statistical uncertainty, or raw event data and sample sizes, will be extracted. Where necessary, alternative summary statistics will be converted to a common metric to facilitate quantitative synthesis;"

Pages 14-15, Data Synthesis:

- "To evaluate the relationship between ~~the severity of~~ anterior chamber inflammation ~~in active anterior uveitis~~ and corneal immunological features in

	anterior uveitis, a meta-analysis will be performed if more than one study reports relevant quantitative ~~correlation~~ data suitable for comparison. Studies will be grouped by anterior chamber inflammation status, dichotomised into active inflammation (SUN > 0.5+) and inactive inflammation (SUN ≤ 0.5+). Studies will be included in the meta-analysis only if anterior chamber inflammation is reported using the SUN criteria for anterior chamber cells ~~and flare~~,[8] or if alternative grading systems can be converted to equivalent SUN grades. If the grading method is unclear or cannot be reliably converted to the SUN scale, the study will be excluded from the quantitative synthesis of this outcome. ~~If needed, correlation coefficients (r) will be transformed to Fisher's z-values before meta-analysis, using either a fixed-effect model (when data are available for three or fewer studies) or random-effects model (when data are available for more than three studies)~~."
8. Sensitivity and subgroup analyses Page 14; The planned sensitivity and subgroup analyses are generally appropriate. Please clarify whether the sensitivity analysis excluding studies at high risk of bias will be based on overall risk of bias only, or on specific domains such as participant selection.<br><br>The Subgroup Analysis section lists several possible subgroup variables, including uveitis subtype, treatment type, participant age group, measurement tool/device, tissue location and contralateral-eye effects. Please clarify which subgroup analyses are considered a priori and most directly linked to the review objectives, and how	The manuscript text has been revised to specify that only the overall risk of bias will be considered in the context of the sensitivity analysis.<br>• **Page 15, Sensitivity Analysis:** "A sensitivity analysis will be performed for the primary outcome measures if sufficient data are available. This analysis will assess the impact of excluding studies that were judged to have a high risk of bias overall ~~or specifically due to factors related to bias in the selection of the study populations~~."<br><br>All the proposed subgroup analyses have been specified a priori, based on factors deemed by our team to be clinically and methodologically relevant to the outcome measures. If there are few studies, and methodological heterogeneity, such findings will be interpreted cautiously and

subgroup findings will be interpreted given the likely small number of studies and expected methodological heterogeneity.	appropriately considered hypothesis-generating rather than confirmatory.
9. Suggested summary table Methods section (overall); Given the number of review questions, outcomes, comparators and planned analyses, the Methods section would be easier to follow if the authors included a summary table linking each review objective/question to the relevant exposure or comparison, comparator group, outcomes of interest, outcome measures, planned synthesis method, and relevant subgroup analyses. At present, the protocol includes several related but distinct questions, including active or historical anterior uveitis versus healthy controls, differences between anterior uveitis subtypes, associations with anterior chamber inflammation severity, and pre- versus post-treatment changes. These questions involve different comparators and different types of analysis, which can be difficult to follow when presented only in text. A summary table would improve clarity, reproducibility, and alignment between the objectives, outcomes and analysis plan.	Further to the reviewer's suggestion, a new summary table, linking the review objectives, exposures, comparators, outcome measures and planned analytical approaches has been created, and is included as Supplementary Material 3.<br>• **Page 9, Outcome measures:** "A table summarising the review objectives, exposures, comparators, outcome measures and planned analytical approaches is provided in Supplementary Material 3."
10. Discussion and limitations Pages 14-16; The Discussion is generally appropriate for a protocol and clearly explains the potential importance of synthesising evidence on corneal and tear film immunological features in anterior uveitis. However, the limitations section could be strengthened.<br><br>In addition to the stated limitations around exclusion of grey literature, heterogeneity of investigative techniques, and variability in	The Discussion section has been revised in alignment with the reviewer's suggestions:<br>• **Page 16-17, Discussion:** "A possible limitation is the exclusion of grey literature and unpublished works. This is a conscious decision to minimise the inclusion of non-peer-reviewed evidence; however, it is acknowledged that this may increase the risk of publication bias. Restricting studies to those published in English may also introduce language bias… This planned systematic review and meta-analysis will comprehensively evaluate

diagnostic definitions, please acknowledge the English-language restriction as a potential source of language bias. Please also balance the statement about excluding grey literature: while this may reduce reliance on non-peer-reviewed sources, it may also increase the risk of publication bias.<br><br>Finally, please ensure that statements about the clinical potential of corneal or tear film biomarkers remain appropriately cautious. This review can identify and synthesise candidate immunological features, but further validation would be needed before these features could be considered clinically useful biomarkers.	the available evidence on human corneal and tear film immunological features in anterior uveitis ~~as a foundation to inform future biomarker research~~. While the review may identify candidate immunological features associated with anterior uveitis, further validation in well-designed prospective studies will be required before any identified features could be considered clinically useful biomarkers. Nevertheless, improving our understanding of ocular surface immunological features may provide novel insight into anterior segment pathology in anterior uveitis, and lay the groundwork for future research aimed at improving diagnosis, monitoring and therapy in anterior uveitis."

VERSION 2 - REVIEW

Reviewer **1**

Name **Nouraeinejad, Ali**

Affiliation **Department of Clinical Ophthalmology, University College London**

Date **22-Jul-2026**

COI

Thank you.

VERSION 2 - AUTHOR RESPONSE

Reviewer	Comment	Authors' response
Reviewer 1	Thank you.	We thank Reviewer 1 for reviewing our revised manuscript and acknowledge that no further changes were requested.
