# Supplementary material for: Corneal and tear film immunology in anterior uveitis: protocol for a systematic review and meta-analysis
Source: BMJ Open. 2026 Aug 12;16(8):e121276. doi: 10.1136/bmjopen-2026-121276 (PMC13475445; doi:10.1136/bmjopen-2026-121276)
Supplement: Supplementary file 3 [file bmjopen-16-8-s002.docx]

**SUPPLEMENTARY MATERIAL 2** - Corneal and tear film immunology in anterior uveitis: protocol for a systematic review and meta-analysis

**Ovid MEDLINE Search Strategy**

| 1 | Exp uveitis/ or (uveitis or iritis or iridocyclitis).mp. |
| --- | --- |
| 2 | Tears/ or (tear film or tear fluid or tears or lacrimal fluid).mp. |
| 3 | 1 and 2 |
| 4 | Exp cornea/ or cornea*.mp. |
| 5 | Exp leukocytes/ or (keratic or leukocyte* or dendritic cell* or macrophage* or T cell* or neutrophil* or lymphocyte* or immune cell* or immunologic* or Langerhans cell* or immunocompetent cell*).mp. |
| 6 | Microscopy, Confocal/ or (confocal microscop* or confocal laser scanning microscopy).mp. |
| 7 | 5 or 6 |
| 8 | 4 and 7 |
| 9 | 1 and 8 |
| 10 | 3 or 9 |

**Ovid Embase Search Strategy**

| 1 | Exp uveitis/ or exp iridocyclitis/ or (uveitis or iritis or iridocyclitis).mp. |
| --- | --- |
| 2 | Exp tear film/ or exp lacrimal fluid/ or (tear film or tear fluid or tears or lacrimal fluid).mp. |
| 3 | 1 and 2 |
| 4 | Exp cornea/ or cornea*.mp. |
| 5 | Exp leukocytes/ or (keratic or leukocyte* or dendritic cell* or macrophage* or T cell* or neutrophil* or lymphocyte* or immune cell* or immunologic* or Langerhans cell* or immunocompetent cell*).mp. |
| 6 | Exp confocal microscopy/ or (confocal microscop* or confocal laser scanning microscopy).mp. |
| 7 | 5 or 6 |
| 8 | 4 and 7 |
| 9 | 1 and 8 |
| 10 | 3 or 9 |
| 11 | limit 10 to conference abstract status |
| 12 | 10 not 11 |

**Cochrane CENTRAL Search Strategy**

| 1 | Exp uveitis/ or (uveitis or iritis or iridocyclitis).mp. |
| --- | --- |
| 2 | Tears/ or (tear film or tear fluid or tears or lacrimal fluid).mp. |
| 3 | 1 and 2 |
| 4 | Exp cornea/ or cornea*.mp. |
| 5 | Exp leukocytes/ or (keratic or leukocyte* or dendritic cell* or macrophage* or T cell* or neutrophil* or lymphocyte* or immune cell* or immunologic* or Langerhans cell* or immunocompetent cell*).mp. |
| 6 | Microscopy, Confocal/ or (confocal microscop* or confocal laser scanning microscopy).mp. |
| 7 | 5 or 6 |
| 8 | 4 and 7 |
| 9 | 1 and 8 |
| 10 | 3 or 9 |
