# Supplementary material for: Corneal and tear film immunology in anterior uveitis: protocol for a systematic review and meta-analysis
Source: BMJ Open. 2026 Aug 12;16(8):e121276. doi: 10.1136/bmjopen-2026-121276 (PMC13475445; doi:10.1136/bmjopen-2026-121276)
Supplement: Supplementary file 4 [file bmjopen-16-8-s003.docx]

**SUPPLEMENTARY MATERIAL 3** - Corneal and tear film immunology in anterior uveitis: protocol for a systematic review and meta-analysis

**Methods Summary Table**

| **Review objective** | **Exposure** | **Comparator** | **Outcome measures** | **Effect measures** | **Planned synthesis / analyses^** |
| --- | --- | --- | --- | --- | --- |
| (1°) To identify and characterise the immunological features of the human cornea and tear film in eyes with anterior uveitis (active or inactive) and healthy (control) eyes | Anterior uveitis:  (i) Active  (ii) Inactive   a) Remission b) Quiescent | Healthy controls or  fellow-eye controls | Primary:   - Corneal immune cell counts/density - Tear cytokine and chemokine levels   Secondary:   - Corneal immune cell morphology* - Tear non-cytokine and non-chemokine mediator levels | Mean difference (or standardised mean difference) between groups | Meta-analysis, where ≥2 studies report the same quantitative outcome |
| (2°) To examine if there are differences in corneal immunological features and tear mediator levels for different subtypes of anterior uveitis during active disease | Anterior uveitis subtype  (e.g., infectious) | Other reported anterior uveitis subtype (e.g., non-infectious) | - Corneal immune cell features* - Tear mediator levels | Mean difference (or standardised mean difference) between groups  Odds ratio | Meta-analysis where ≥2 studies report the same quantitative outcomes for each subtype  Subgroup analyses when ≥3 studies report quantitative data on the same subtype |
| (2°) To determine the association between anterior chamber inflammation and corneal immunological features in anterior uveitis | Active anterior chamber inflammation | Inactive anterior chamber inflammation | Corneal immune cell features* | Mean difference (or standardised mean difference) between groups  Odds ratio | Meta-analysis where ≥2 studies report quantitative correlation data |
| (2°) To assess for differences in corneal immunological features and tear mediator levels pre- and post- treatment of anterior uveitis | Treatment / intervention | Pre-treatment (of the same eye/ participant) | - Corneal immune cell features* - Tear mediator levels | Within-group mean change between baseline and follow-up | Meta-analysis where ≥2 studies report quantitative pre- vs post-treatment data on the same outcome  Subgroup analyses when ≥3 studies report quantitative data on the same treatment type |

*Corneal immune cell features include counts/density and/or morphology, stratified by tissue layer (epithelium, stroma, endothelium [keratic precipitates]).

^For primary and secondary outcomes where a meta-analysis is not possible due to insufficient data or significant heterogeneity, a narrative synthesis will be provided.
